# Linking Embodiment, Simulator Sickness, and EEG Activity During XR–BCI Use: A Single-Participant Case Study

**DOI:** 10.3390/life16081352

**Published:** 2026-08-17

**Authors:** Diogo João Tomás, Miguel Pais-Vieira, Carla Pais-Vieira

**Affiliations:** 1 Department of Physiotherapy, Escola Superior de Saúde do Alcoitão, 2649-506 Alcabideche, Portugal; diogo.tomas@essa.scml.pt; 2 iBiMED—Instituto de Biomedicina, Departamento de Ciências Médicas, Universidade de Aveiro, 3810-193 Aveiro, Portugal; miguelpaisvieira@ua.pt; 3 Center for Interdisciplinary Research in Health (CIIS), Faculty of Health Sciences and Nursing, Universidade Católica Portuguesa, 4160-005 Porto, Portugal

**Keywords:** embodiment, electroencephalography, virtual reality, brain–computer interface, simulator sickness, spinal cord injury

## Abstract

**Background:** Subjective experience is increasingly recognised as an important component of brain–computer interface (BCI) performance in extended reality (XR) environments. Although embodiment and simulator sickness are known to influence user experience, their relationships with cortical activity during XR–BCI operation remain poorly understood. Building upon our previous investigations of embodiment and simulator sickness in XR–BCIs, the present study examined whether these subjective dimensions are associated with distinct neurophysiological patterns during repeated XR–BCI use in a participant with chronic spinal cord injury (SCI). **Methods:** Seventeen XR–BCI sessions performed by a participant with chronic complete SCI were analysed. Bayesian correlation analyses examined associations among embodiment, simulator sickness, BCI performance, and EEG activity. Multiple linear regression was used to identify variables independently associated with sensorimotor beta activity, and the robustness of the regression findings was evaluated using bootstrap estimation and leave-one-out sensitivity analyses. **Results:** Bayesian analyses identified two principal patterns of association. Sense of embodiment was positively associated with frontal theta activity (F3), whereas simulator sickness showed a negative association with sensorimotor beta activity (C3–C4). As expected, classifier acquisition accuracy was strongly associated with subsequent BCI performance. Multiple regression demonstrated that simulator sickness was the only variable independently associated with C3–C4 beta activity after accounting for embodiment and BCI performance. This association remained robust following bootstrap estimation and leave-one-out sensitivity analyses. **Conclusions:** Although limited to a single participant, these findings suggest that different dimensions of subjective experience during XR–BCI operation are associated with partially distinct neurophysiological correlates. In particular, simulator sickness was the variable most consistently associated with sensorimotor beta activity across all analyses. These findings provide a foundation for future longitudinal investigations of the neural mechanisms linking subjective experience and cortical dynamics during XR–BCI use.

## 1. Introduction

The sense of embodiment (SoE) refers to the subjective experience of owning, controlling, and being located within a body, emerging from the dynamic integration of multisensory signals with prior beliefs and predictions about bodily states [1,2,3,4]. Rather than a static percept, embodiment is increasingly understood as an inferential process shaped by the continuous reconciliation of sensory evidence and internal models, a view supported by classic paradigms such as the Rubber Hand Illusion [5] and by neuroimaging studies on the role of frontoparietal networks in body representation [6,7].

Extended-reality (XR) systems combining Virtual Reality (VR) and Brain–Computer Interfaces (BCI) provide a powerful framework for studying embodiment under conditions of active control and sensorimotor dissociation. VR enables precise manipulation of sensory contingencies, whereas BCI systems decode neural activity to control virtual avatars, allowing researchers to probe ownership, agency, and self-location in ecologically valid yet experimentally controlled environments [8,9]. Beyond their experimental relevance, XR–BCI systems have gained increasing attention in neurorehabilitation. In such contexts, multimodal XR–BCI systems can reconstruct coherent sensorimotor loops by combining visual, tactile, auditory, and motor-intent signals, and have been associated with changes in sensorimotor activity, modulation of body representation, reductions in neuropathic pain, and alterations in neural dynamics [10,11,12,13,14,15,16].

Despite these advances, the neurophysiological mechanisms linking subjective experience to cortical activity during XR–BCI operation remain incompletely understood. Most embodiment studies treat SoE as an outcome measure, comparing experimental conditions rather than examining within-subject temporal variability [2,17,18,19]. This approach overlooks meaningful fluctuations across sessions—fluctuations that may reflect dynamic changes in brain–body integration, particularly in clinical populations such as SCI, where altered sensory input and increased reliance on top-down processes may reshape embodiment dynamics [11,15,20].

Electroencephalography (EEG) is well suited to capture these dynamics due to its millisecond-level temporal precision [21,22,23,24,25]. Embodiment-related activity has been associated with distributed frontoparietal and sensorimotor networks, with consistent modulation of alpha (mu) rhythms in sensorimotor contexts [26,27]. Evidence for beta and gamma oscillations is more variable [18,28,29], and theta activity has also been associated with attentional allocation and cognitive control, rather than exclusively with embodiment [30]. In motor-imagery BCIs, reduced alpha and increased theta power have been linked to BCI illiteracy [31], potentially reflecting differences in sensorimotor integration and embodiment-related processes [32]. Recent work suggests that parietal alpha dynamics may contribute to body ownership through temporal integration of multisensory signals [33].

Previous studies using the same multimodal XR–BCI system reported sustained embodiment across repeated sessions in a participant with chronic complete SCI [12], and prolonged use was later associated with reductions in neuropathic pain, neurophysiological changes, and the emergence of rhythmic lower-limb movements [13]. However, the relationships between embodiment, cybersickness, neural activity, and BCI performance remain poorly understood. Recent work in healthy participants showed that embodiment is strongly influenced by user-experience factors, including simulator sickness (Tomás et al., submitted), suggesting the possibility that fluctuations in subjective experience may contribute to variability in BCI control and associated neural processes.

The present study constitutes a secondary analysis of data acquired during the longitudinal XR–BCI protocol previously reported by Pais-Vieira et al. [12,13] and introduces no structural or technical modifications to the system. Its contribution is analytical and mechanistic rather than technical: it examines previously uninvestigated session-to-session relationships among embodiment, simulator sickness, BCI performance, and motor-imagery EEG activity. Rather than focusing on a single neural marker of embodiment, we investigated how subjective experience, neural activity, and task performance covaried over time, with particular attention to the complementary associations involving frontal theta activity and sensorimotor beta rhythms. This approach aims to clarify how experiential and neurophysiological states are associated during XR–BCI interaction and to provide a foundation for future mechanistic and translational studies.

Given the single-participant design, the present study is intended as an exploratory, hypothesis-generating investigation of neural mechanisms underlying XR–BCI interaction. Rather than aiming for population-level inference, the goal is to characterize associations across repeated XR–BCI sessions between subjective experience and EEG activity in a clinical context where longitudinal data are rare. This approach complements our recent findings in healthy participants—where simulator sickness emerged as the strongest factor associated with embodiment—and extends that framework to a spinal cord injury case. By examining session-to-session covariation across behavioral and neural measures, the study addresses the gap identified in our systematic review [34] that no prior work has systematically examined the independent contribution of subjective experience to embodiment or BCI performance in XR-BCI systems.

## 2. Materials and Methods

### 2.1. Study Design and Participant

**Figure 1:**
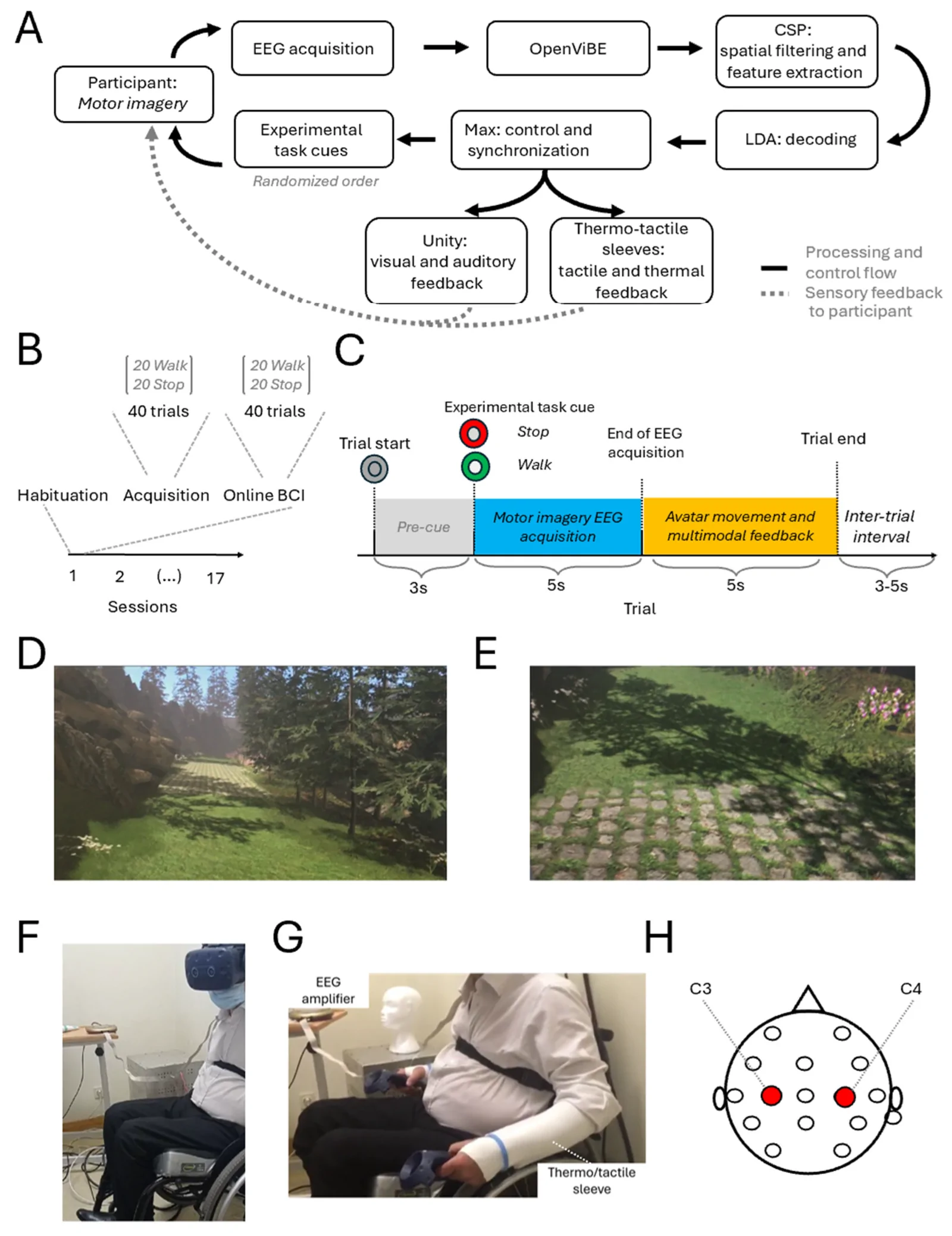
XR–BCI architecture, experimental protocol, virtual environment, and equipment. (**A**) Closed-loop architecture of the XR–BCI system. Motor-imagery EEG signals were acquired and processed online in OpenViBE using common spatial patterns (CSP) for spatial filtering and feature extraction, followed by linear discriminant analysis (LDA) decoding. Max controlled the randomized experimental task cues and coordinated feedback delivery through Unity and the thermo-tactile sleeves. Solid arrows indicate processing and control flow, whereas dotted arrows indicate sensory feedback delivered to the participant. Avatar movement and the associated tactile, thermal and auditory feedback were synchronized with the experimental task cues and classifier-related outcome (auditory feedback). (**B**) Organization of the 17 experimental sessions. Each session comprised 40 acquisition trials followed by 40 online BCI trials, with each phase including 20 “Walk” and 20 “Stop” trials presented in randomized order. (**C**) Individual-trial timeline, comprising a 3 s pre-cue period, a 5 s motor-imagery EEG acquisition period, a 5 s avatar movement and multimodal feedback period, and a variable 3–5 s inter-trial interval. (**D**,**E**) Representative views of the virtual forest environment as presented to the participant. (**F**) Participant wearing the head-mounted display during an experimental session. (**G**) Experimental setup showing the EEG amplifier and the thermo-tactile sleeves positioned on the participant’s forearms. (**H**) Schematic representation of the EEG montage, highlighting the C3 and C4 electrodes used to calculate the sensorimotor beta-activity difference.

This study employed a longitudinal single-case repeated-session design to investigate the relationships among subjective experience, brain–computer interface (BCI) performance, and electroencephalographic (EEG) activity during repeated extended reality (XR)–BCI sessions (see Figure 1). The participant was recruited at Hospital Senhora da Oliveira, Portugal, provided written informed consent prior to participation, and the study was approved by the local Ethics Committee (CES–Hospital Senhora da Oliveira, No. 15/2020) in accordance with the Declaration of Helsinki. Data collection took place during the COVID-19 pandemic, which substantially restricted the recruitment of additional participants with spinal cord injury and the conduct of repeated in-person experimental sessions. Consequently, the study was conducted as an intensive longitudinal single-participant investigation.

The participant was a 52-year-old male with a complete spinal cord injury (SCI) at the T4 neurological level who had remained clinically stable for 32 years. He had previously participated in studies using the same multimodal XR–BCI system [12,13], which has been described in detail elsewhere [35].

The broader longitudinal protocol was conducted under the clinical oversight of specialists in Physical and Rehabilitation Medicine and Neurology. Serial neurological examinations were performed throughout the protocol in accordance with the International Standards for Neurological Classification of Spinal Cord Injury (ISNCSCI; commonly referred to as the ASIA examination). These assessments included sensory testing across the relevant dermatomes, motor testing of key muscle groups, and assessment of sacral sparing to determine the neurological level of injury and AIS grade. Examinations were conducted by the medical specialists or by medical residents under their supervision. The participant remained classified as T4 AIS A throughout the study, as reported previously [12,13].

Experimental sessions were conducted by trained researchers, who supervised participant positioning, equipment calibration, EEG signal quality, operation of the XR–BCI system, and monitoring of participant comfort and possible adverse effects. At the beginning and end of each session, a brief interview was conducted to assess medication use, sleep quality, fatigue, attention, emotional state, motivation, pain, and general clinical status. Relevant deviations from the participant’s usual condition were recorded. Medication remained unchanged throughout the study, and no persistent changes in the other monitored factors were identified.

#### Top of Form

For the present study, 17 XR–BCI sessions conducted over approximately six months were selected based on data completeness and consistency of the experimental protocol. These sessions represented a continuous period of approximately weekly assessments, thereby minimising interruptions in training and adaptation. The complete longitudinal dataset included an additional two-month interruption, which was excluded from the present analyses to maximise temporal consistency across sessions.

The present analyses were performed on a subset of the longitudinal dataset reported in our previous investigations. Whereas earlier studies primarily examined longitudinal changes in embodiment, simulator sickness, neuropathic pain, and neurophysiological measures [12,13], the present study focused on the associations among embodiment, simulator sickness, BCI performance, and cortical activity across repeated XR–BCI sessions using Bayesian correlation and multivariable regression analyses. Rather than investigating longitudinal changes over time, the aim was to determine whether session-to-session variations in subjective experience were associated with corresponding variations in neural activity and BCI performance.

Because the dataset consisted of repeated observations from a single participant, all statistical analyses were interpreted as exploratory within-participant analyses rather than as supporting population-level inference. To strengthen the robustness of the principal findings, multivariable regression results were complemented by regression diagnostics, bias-corrected and accelerated (BCa) bootstrap estimation (5000 resamples), and leave-one-out sensitivity analyses.

### 2.2. Apparatus

The system used in this study consisted of a multimodal brain–computer interface (BCI) integrated with a virtual reality (VR) environment. The VR setup included a head-mounted display with integrated headphones for visual and auditory stimulation, together with two handheld controllers used during the habituation phase (HTC Vive Pro Eye, HTC Corporation, New Taipei City, Taiwan). The virtual environment was presented from a first-person perspective.

Multisensory feedback was delivered through a pair of custom-developed thermo-tactile sleeves capable of providing both tactile and thermal stimulation. Tactile feedback was generated by six independently controlled vibrotactile actuators embedded in each sleeve, whereas thermal feedback was delivered within a temperature range of 18–35 °C. These feedback modalities were synchronised with the avatar’s movements so that simulated foot–ground contact was conveyed to the participant’s forearms.

Neural activity was recorded using a 16-channel EEG system (V-Amp amplifier with actiCAP electrodes; Brain Products GmbH, Gilching, Starnberg, Germany). EEG signals were acquired and processed online using OpenViBE. Signal quality was verified before each session by visual inspection and standard physiological procedures, including eye opening and closing, mastication, and brief eyes-closed recordings (~10 s).

Both the acquisition and online decoding phases began with a 20–30 s resting baseline. EEG preprocessing and BCI calibration followed the standard OpenViBE motor imagery pipeline, including common spatial pattern (CSP) filtering and training of a two-class linear discriminant analysis (LDA) classifier. The trained classifier was subsequently used for real-time decoding during the online BCI phase.

The software responsible for synchronising and controlling the complete XR–BCI system was developed in Max (Cycling ‘74, San Francisco, CA, USA), whereas the virtual environments were developed in Unity (Unity Technologies, San Francisco, CA, USA).

### 2.3. Experimental Procedure

Each session lasted approximately 70–90 min and followed a structured protocol consisting of three main phases. During the habituation phase, the participant was introduced to the VR environment and interacted with the avatar using hand controllers, allowing adaptation to the virtual scenario and adjustment of the equipment.

During the EEG baseline and acquisition phase, a baseline period of 20–30 s was recorded while the participant remained in the virtual environment. This was followed by a motor imagery task in which visual cues instructed the participant to imagine walking (“Walk”) or remaining still (“Stop”). Each session included 40 acquisition trials, followed by 40 online BCI trials, with 20 “Walk” and 20 “Stop” trials in each phase.

During the neural decoding phase, EEG signals were processed using a motor imagery classifier. Auditory feedback regarding decoding accuracy was provided, while avatar movement remained visually congruent with task cues. The participant performed all sessions in a seated position and experienced synchronized visual, auditory, and tactile feedback corresponding to the avatar’s movements.

### 2.4. Embodiment Assessment

An adapted embodiment questionnaire was utilized to systematically evaluate the participant’s subjective experience of embodiment during the motor imagery training sessions combined with immersive virtual reality. This questionnaire was an adaptation of the avatar embodiment questionnaire designed by Peck and Gonzalez-Franco [36], which originally comprises nine items assessing three theoretical domains: sense of ownership, sense of agency and sense of self-location. The adapted questionnaire was applied at the end of each session to capture the participant’s immediate responses to the embodiment experiences.

### 2.5. EEG Processing

Electroencephalography (EEG) data were recorded using a 16-channel system (actiCAP, Brain Products GmbH, Gilching, Germany), following the international 10–20 system, with electrodes distributed across frontal, central, parietal, temporal, and occipital regions [37,38]. Signals were sampled at 1000 Hz. Preprocessing and subsequent feature extraction followed standard EEG and related biomedical signal-processing principles [37,38,39,40]. Preprocessing included band-pass filtering (0.5–70 Hz), 50 Hz notch filtering, and ocular artifact correction using the Gratton and Coles method. All preprocessing steps, filtering parameters, and artifact-rejection thresholds were applied identically across all recording sessions, and no session-specific adjustments were performed.

Offline spectral analysis was performed using fast Fourier transform (FFT), and power was extracted for the following frequency bands: delta (0.5–4.5 Hz), theta (4.5–8.5 Hz), alpha (8.5–13.5 Hz), beta (13.5–30 Hz), and gamma (30–45 Hz), with the latter corresponding to the low-gamma range as defined in previous studies. Spectral power values were z-score normalized separately for each EEG channel.

For each session, mean spectral power was computed for each frequency band and electrode, and subsequently averaged within predefined scalp regions (frontal, central, parietal, and occipital). Although the EEG recordings included multiple channels and frequency bands, the primary analyses were defined a priori to focus on F3 theta and C3–C4 beta because of their strong theoretical relevance to multisensory conflict, embodiment, and motor imagery processing. A supplementary analysis of the alpha band at C3–C4 was also conducted using the same statistical pipeline; however, no meaningful associations were observed, and the null model was consistently favored.

Motor imagery decoding followed a two-class pipeline adapted from OpenViBE. Sixteen channels were spatially filtered using a single pair of Common Spatial Patterns (CSP), yielding two components optimized for discriminating “Walk” vs. “Not Walk” mental states. CSP-filtered signals were band-pass filtered between 8 and 24 Hz and segmented within a 5 s post-cue window. Mean band-power features from each segment were used to train a participant-specific Linear Discriminant Analysis (LDA) classifier. Performance was evaluated using 5-fold cross-validation during calibration before deployment for real-time decoding.

### 2.6. Statistical Analysis

**Figure 2:**
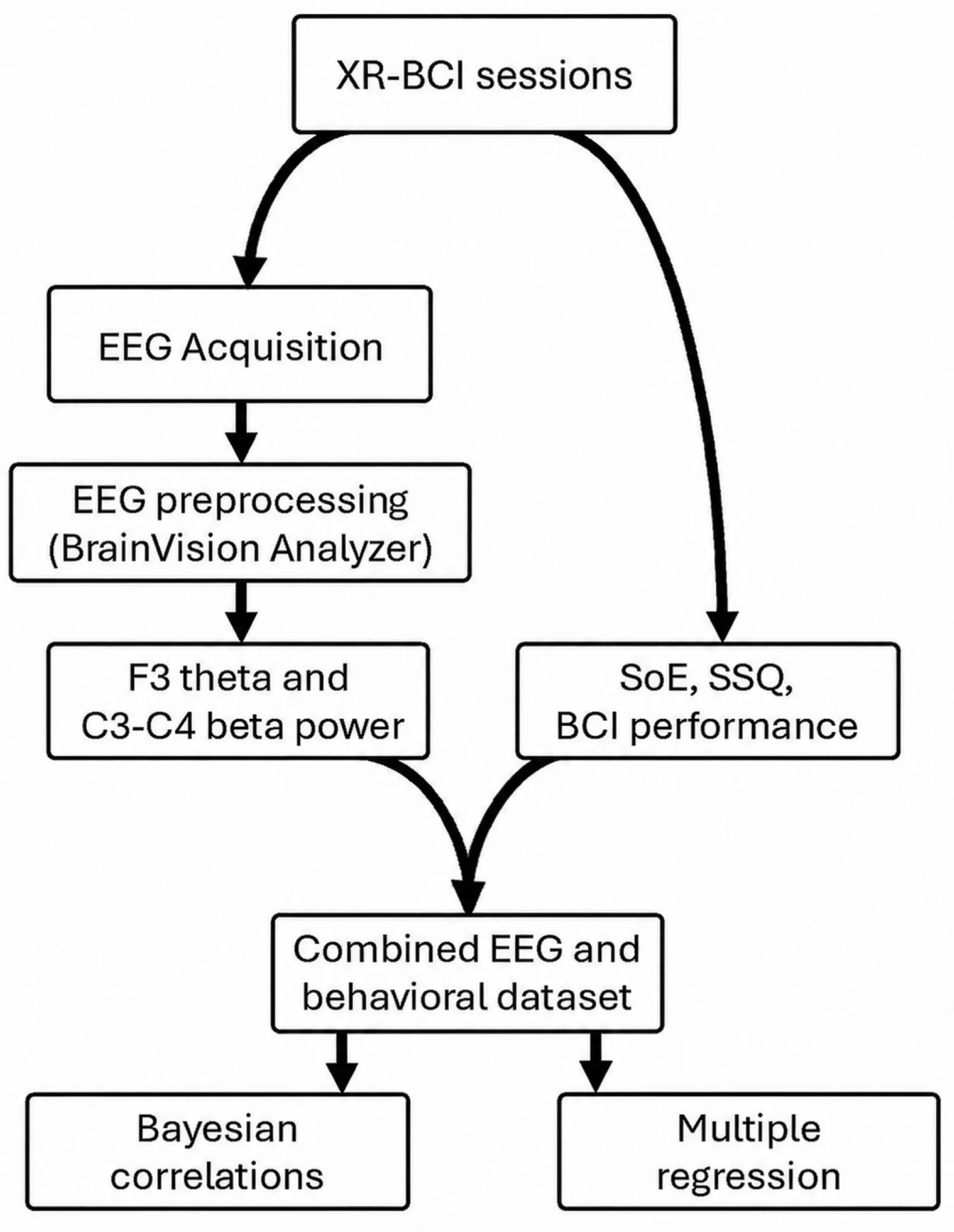
Offline EEG and statistical analysis workflow. EEG data acquired during the XR–BCI sessions were preprocessed in BrainVision Analyzer. Frontal theta power at F3 and sensorimotor beta power at C3–C4 were then integrated with sense of embodiment (SoE), Simulator Sickness Questionnaire (SSQ), and BCI-performance measures for Bayesian correlation and multiple regression analyses.

**Figure 3:**
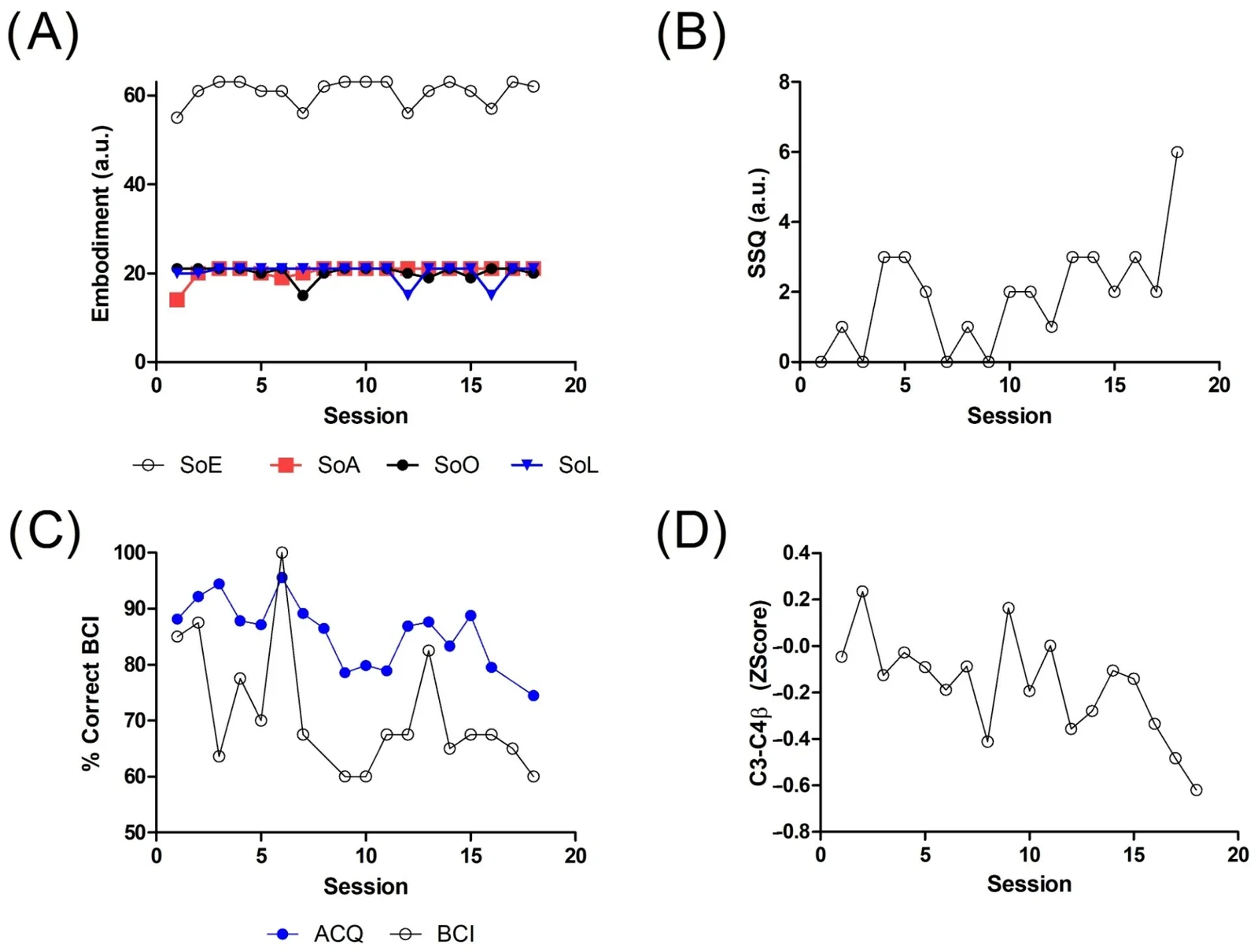
Overview of the behavioural and neurophysiological variables across the 17 XR–BCI sessions. (**A**) Session-by-session scores for the global sense of embodiment (SoE) and its three components: sense of agency (SoA), sense of ownership (SoO), and sense of self-location (SoL). Overall embodiment remained consistently high, with only modest fluctuations across sessions. (**B**) Simulator Sickness Questionnaire (SSQ) scores across sessions. Simulator sickness remained generally low but exhibited session-to-session variability. (**C**) Brain–computer interface performance during the acquisition (ACQ) and online BCI control phases. Acquisition accuracy was consistently higher than online BCI performance, although both measures varied across sessions. (**D**) Session-by-session C3–C4 beta activity (13.5–30 Hz; z-score normalized) during motor imagery. Sensorimotor beta power showed substantial variability across sessions without a consistent temporal trend. These descriptive data provide the behavioural and neurophysiological context for the Bayesian correlation and multivariable analyses presented in Figure 4. In panels (**B**,**D**), open circles represent individual-session values, and connecting lines are included to illustrate session-to-session variation.

Given the single-case repeated-session design, statistical analyses focused on the associations among subjective experience, BCI performance, and cortical activity across sessions (Figure 2 and Figure 3A–D). Because observations consisted of repeated sessions from a single participant, all statistical analyses were interpreted as exploratory within-participant analyses, and no population-level inference was intended.

**Figure 4:**
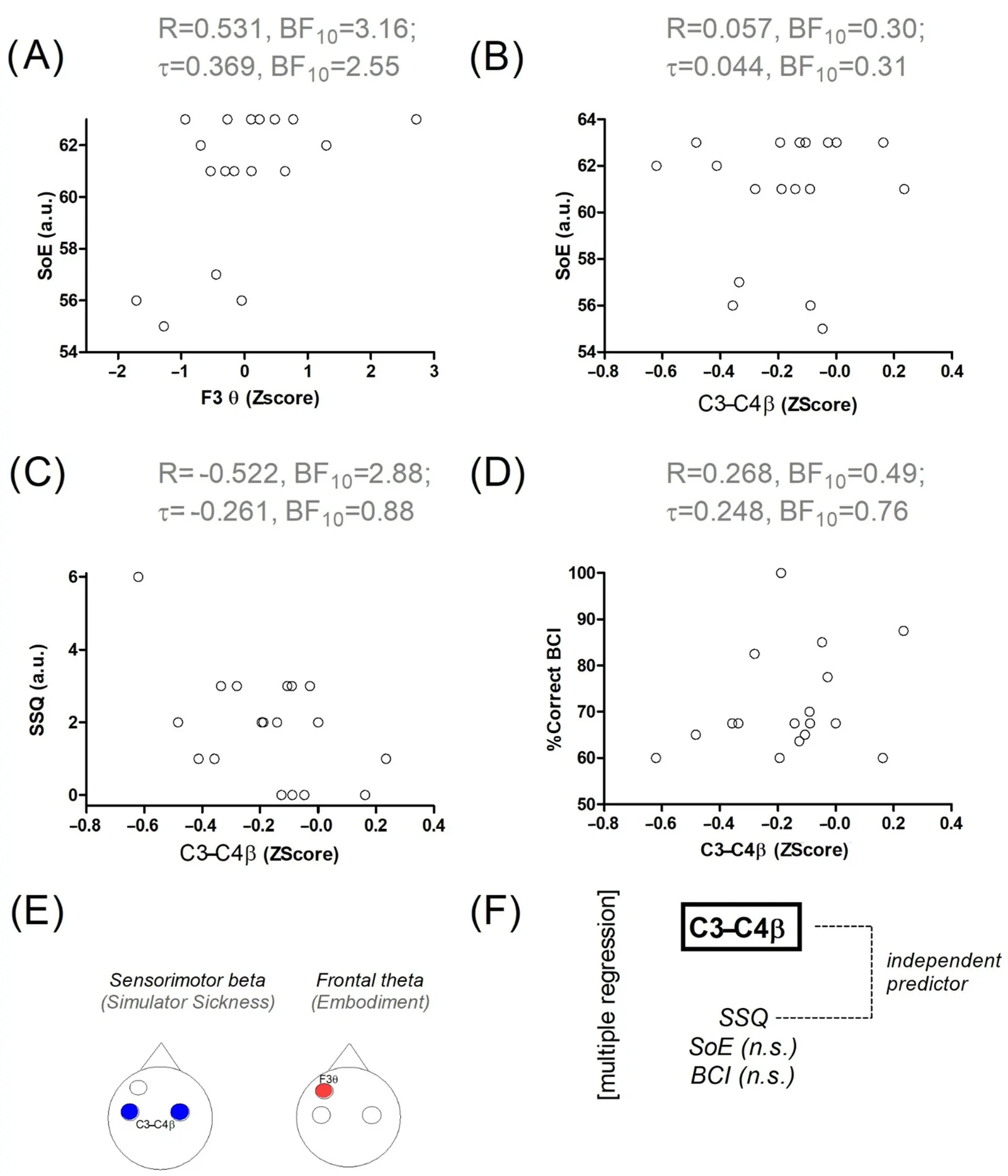
Bayesian associations and multivariable model relating subjective experience, BCI performance, and cortical activity during repeated XR–BCI sessions. (**A**–**D**) Scatterplots showing the principal Bayesian correlations identified across the 17 experimental sessions. (**A**) Positive association between sense of embodiment (SoE) and frontal theta activity (F3). (**B**) No evidence of an association between SoE and sensorimotor C3–C4 beta activity. (**C**) Negative association between simulator sickness (SSQ) and C3–C4 beta activity. (**D**) No evidence of an association between BCI performance and C3–C4 beta activity. Pearson correlation coefficients (r), Kendall’s tau (τ), and corresponding Bayes factors (BF_10_) are shown for each comparison. (**E**) Conceptual summary of the observed associations, illustrating the association of embodiment with frontal theta activity and simulator sickness with sensorimotor beta activity. (**F**) Schematic representation of the multiple linear regression analysis illustrating that simulator sickness emerged as the only independent predictor of C3–C4 beta activity after accounting for embodiment and BCI performance.

Bayesian correlation analyses were first performed to examine pairwise associations between embodiment, simulator sickness, BCI performance, and selected EEG measures. Subsequently, multiple linear regression was conducted to determine whether sensorimotor beta activity was independently associated with simulator sickness after accounting for embodiment and BCI performance.

To evaluate the robustness of the regression findings, bias-corrected and accelerated (BCa) bootstrap estimation (5000 resamples) was applied to the regression coefficients, and leave-one-out sensitivity analyses were additionally performed. Regression diagnostics included assessment of multicollinearity using variance inflation factors (VIFs), residual autocorrelation using the Durbin–Watson statistic, influential observations using Cook’s distance, and visual inspection of residual-versus-fitted and normal Q–Q plots, as well as standardized residuals.

Analyses were conducted using GraphPad Prism (Version 10.4.2; GraphPad Software, San Diego, CA, USA) and JASP (Version 0.18.1; JASP Team, Amsterdam, The Netherlands). Statistical significance was defined as *p* < 0.05 (two-tailed). For Bayesian analyses, evidence was interpreted according to the magnitude of the Bayes factor (BF_10_), following conventional interpretative guidelines [41]. For measurements lacking defined physical units, values are reported in arbitrary units (a.u.).

## 3. Results

Across the 17 XR–BCI sessions, descriptive inspection of the main behavioural and neurophysiological variables revealed stable yet fluctuating patterns (Figure 3, also see Supplementary Figures S1 and S2). Global sense of embodiment remained consistently high, while ownership, agency, and self-location exhibited only modest session-to-session variability (Figure 3A). Simulator sickness scores were generally low but showed meaningful fluctuations across sessions (Figure 3B). Acquisition accuracy was consistently higher than online BCI performance (Figure 3C, also see Supplementary Figures S3 and S4 for details on the processing pipeline), whereas C3–C4 beta activity exhibited marked variability across sessions (Figure 3D). These descriptive observations motivated the subsequent Bayesian correlation, multivariable regression, and robustness analyses.

### 3.1. Bayesian Correlation Analysis

To identify potential associations between embodiment, simulator sickness, BCI performance, frontal activity, and sensorimotor activity, targeted Bayesian correlation analyses were performed across the 17 XR–BCI sessions (Table 1 and Figure 4A–D).

The analyses revealed three principal patterns. First, evidence supported a positive association between sense of embodiment (SoE) and F3 theta activity (Pearson: *r* = 0.531, BF_10_ = 3.16; Kendall: τ = 0.369, BF_10_ = 2.55), indicating that higher embodiment scores were associated with greater frontal theta activity across sessions (Figure 4A). Second, simulator sickness (SSQ) showed evidence of a negative association with C3–C4 beta activity in the Pearson analysis (Pearson: *r* = −0.522, BF_10_ = 2.88), whereas the corresponding Kendall analysis provided weaker evidence (τ = −0.261, BF_10_ = 0.88) (Figure 4C). This pattern suggests that the observed relationship may be better characterised as linear than strictly monotonic. Third, strong evidence supported the expected association between classifier acquisition accuracy and subsequent BCI performance (Pearson: *r* = 0.661, BF_10_ = 10.97; Kendall: τ = 0.521, BF_10_ = 12.28) (Figure 4D).

The remaining targeted comparisons provided little or no evidence for meaningful associations. Specifically, the relationship between SoE and SSQ showed anecdotal or null evidence (Pearson BF_10_ = 0.57; Kendall BF_10_ = 0.42), as did the association between SoE and C3–C4 beta activity (Pearson BF_10_ = 0.30; Kendall BF_10_ = 0.31). Similarly, little evidence supported an association between BCI performance and C3–C4 beta activity (Pearson BF_10_ = 0.49; Kendall BF_10_ = 0.76).

Taken together, these findings are consistent with partially distinct patterns of association, whereby frontal theta activity covaried with subjective embodiment, whereas sensorimotor beta activity was associated with simulator sickness. In contrast, no evidence was found for direct associations between embodiment and sensorimotor beta activity or between BCI performance and sensorimotor beta activity. The strong association between acquisition accuracy and subsequent BCI performance was consistent with the expected relationship between classifier calibration and online BCI control.

Given the observed association between simulator sickness and sensorimotor beta activity, a multivariable regression analysis was subsequently performed to determine whether this relationship remained after accounting for embodiment and BCI performance.

### 3.2. Multivariable Analysis of Sensorimotor Beta Activity

To determine whether sensorimotor beta activity during the BCI phase was independently associated with behavioural and subjective measures, a multiple linear regression was performed using C3–C4 beta power as the dependent variable and simulator sickness (SSQ), sense of embodiment (SoE), and BCI performance as predictors (Figure 4F and Table 2).

The overall regression model was statistically significant (*F*(3,13) = 4.495, *p* = 0.023), explaining 50.9% of the variance in C3–C4 beta activity (*R*^2^ = 0.509; adjusted *R*^2^ = 0.396). Simulator sickness was the only significant independent predictor of C3–C4 beta activity (standardized β = −0.678, *t* = −3.303, *p* = 0.006), with higher SSQ scores being associated with lower sensorimotor beta power. Neither sense of embodiment (standardized β = 0.374, *p* = 0.098) nor BCI performance (standardized β = 0.276, *p* = 0.192) independently contributed to the model.

Diagnostic analyses supported the adequacy of the regression model. No evidence of problematic multicollinearity was observed (VIF range = 1.06–1.17), residuals showed no evidence of significant autocorrelation (Durbin–Watson = 2.423, *p* = 0.431), and inspection of standardized residuals, residual-versus-fitted plots, and Q–Q plots did not reveal substantial deviations from the assumptions of linear regression. No influential observations were identified (maximum Cook’s distance = 0.586), and all standardized residuals remained within ± 2.24.

To evaluate the robustness of the findings, bias-corrected and accelerated (BCa) bootstrap estimation (5000 resamples) and leave-one-out sensitivity analyses were performed. Bootstrap analysis confirmed simulator sickness as the only predictor remaining statistically significant (bootstrap *p* = 0.007). Likewise, sequential exclusion of each session demonstrated that the association between simulator sickness and C3–C4 beta activity remained consistently negative across all leave-one-out models (standardized β range = −0.51 to −0.77). Statistical significance was retained in 16 of the 17 iterations, with only one iteration yielding a non-significant result (*p* = 0.070), indicating that the observed association was robust and was not driven by any single session.

## 4. Discussion

The present study explored the relationships among embodiment, cybersickness, BCI performance, and cortical activity during repeated XR–BCI sessions in a participant with chronic spinal cord injury. This study is a secondary analysis of neurophysiological and subjective data and was not designed to establish clinical efficacy. Bayesian analyses identified two distinct patterns of association: embodiment was associated with frontal theta activity, whereas simulator sickness was associated with reduced sensorimotor beta activity. These findings are consistent with theoretical models of embodiment and multisensory integration [1,2,4] and with previous work on simulator sickness/cybersickness in immersive virtual environments [8,9]. Multiple regression analysis further indicated that simulator sickness was the only variable independently associated with C3–C4 beta power after accounting for embodiment and BCI performance. Importantly, this association remained robust following bootstrap estimation and leave-one-out sensitivity analyses, indicating that it was not driven by any individual session. Together, these findings are consistent with the possibility that different dimensions of subjective XR–BCI experience involve partially distinct cortical processes, highlighting the possibility of partially distinct neurophysiological processes underlying embodiment-related frontal dynamics and cybersickness-related sensorimotor activity.

F3 theta power correlated with embodiment. This pattern aligns with the view of embodiment as a robust but dynamically regulated construct, shaped by the integration of multisensory cues, motor predictions, and contextual factors [1,2,4]. In XR–BCI contexts, where sensory feedback is artificially constructed, stability in SoE may reflect successful reconstruction of a coherent sensorimotor loop despite the participant’s complete SCI [15,20].

However, the presence of local fluctuations suggests that embodiment is not static. Even when average SoE remains high, moment-to-moment or session-to-session variations may reflect subtle changes in internal state, attentional allocation, or multisensory integration demands. This dual structure—stable baseline with dynamic micro-variability—is consistent with inferential models of bodily self-consciousness [4].

Simulator sickness remained low overall but showed meaningful variability across sessions. Prior work in healthy participants demonstrated that simulator sickness can strongly influence embodiment and user experience (Tomás et al., submitted). In XR–BCI contexts, simulator sickness may act as a proxy for multisensory conflict, reflecting discrepancies between visual, vestibular, proprioceptive, and interoceptive cues [8,9]. For individuals with SCI, who rely more heavily on top-down predictions and visual feedback, such conflicts may be particularly salient [11]. The present findings did not provide evidence that simulator sickness was directly associated with embodiment or BCI performance. Instead, its association emerged only at the neurophysiological level, suggesting that multisensory conflict may modulate neural processing without directly affecting behavioural performance.

The principal finding of the present study was that simulator sickness remained the only variable independently associated with C3–C4 beta activity across multivariable, bootstrap, and leave-one-out sensitivity analyses. This finding is consistent with previous evidence reporting an inverse relationship between cybersickness severity and EEG beta activity [42], although the present results further suggest a specific involvement of sensorimotor regions. Within this repeated-session case study, this pattern suggests that multisensory conflict may have exerted a stronger influence on sensorimotor cortical dynamics than either embodied experience or task performance in the XR–BCI context. This interpretation aligns with prior observations: early sessions showed high comfort and stable embodiment [12], extended training was associated with neurophysiological changes and emergent rhythmic movements [13], and embodiment–sickness coupling was observed in healthy participants (Tomás et al., submitted).

In this participant, the observed beta dynamics may be compatible with compensatory mechanisms engaged under increased sensory uncertainty, helping maintain sensorimotor integration when the XR–BCI environment becomes more demanding. This interpretation is tentatively consistent with prior work linking beta rhythms to sensorimotor engagement, predictive coding, and the integration of motor intentions with sensory [26,27,33], although in the present single-participant dataset it should be viewed as an exploratory correspondence rather than a generalizable mechanism.

To evaluate whether these effects generalized across sensorimotor rhythms, we conducted a supplementary analysis of alpha power. Sensorimotor alpha activity did not exhibit modulation by embodiment, cybersickness, or BCI, despite its theoretical relevance in motor imagery and action observation. This dissociation between alpha and beta rhythms may reflect frequency-specific roles in sensorimotor integration. Beta oscillations are closely linked to predictive motor processing and the updating of internal models under sensory conflict, whereas alpha rhythms are more associated with motor preparation and attentional gating. The absence of alpha modulation, combined with robust beta effects, suggests that the XR–BCI captured the expected sensorimotor dynamics for avatar control while revealing that only beta power was sensitive to multisensory uncertainty in this embodiment context.

Although the present analyses do not test interaction effects directly, in this case study the pattern of results could be interpreted as compatible with a coupled dynamical relationship between embodiment, neural activity, and performance. This possibility remains speculative and would require confirmation in larger samples. Sensorimotor beta activity appeared particularly sensitive to fluctuations in cybersickness, suggesting that multisensory conflict may influence how motor-imagery networks operate during XR–BCI use. This perspective helps reconcile why embodiment remained relatively stable while neural and experiential variables fluctuated across sessions: the system may adapt to changing sensory conditions to maintain a coherent sense of self and stable task engagement.

From a predictive-coding perspective, the present pattern of results may reflect how the brain adjusts the weighting of sensory evidence and motor predictions under varying levels of multisensory conflict. Predictive-processing models propose that the sense of embodiment arises from minimizing prediction errors between expected and incoming sensory signals [4]. Although the current analyses do not test interaction effects, the association between cybersickness and reduced sensorimotor beta activity raises the possibility that multisensory conflict influences how motor-imagery networks operate during XR–BCI use. Under higher sensory uncertainty, the system may rely more heavily on internally generated motor predictions to maintain coherent sensorimotor integration. This interpretation aligns with inferential models of bodily self-consciousness and provides a computational perspective on why sensorimotor rhythms may fluctuate under conditions of increased sensory conflict.

From a clinical perspective, these findings suggest that routine monitoring of simulator sickness may provide a practical behavioural indicator of neurophysiological state during prolonged XR–BCI use, although replication in larger longitudinal cohorts is required before clinical implementation can be considered. Nevertheless, given the exploratory repeated-session single-participant design, these mechanistic interpretations should be considered hypothesis-generating and require confirmation in larger longitudinal studies.

### 4.1. Limitations

Several limitations should be acknowledged. First, this study is based on a single participant with chronic spinal cord injury, which limits the generalizability of the findings to broader SCI and BCI populations. The recruitment of additional participants and the conduct of repeated in-person experimental sessions were substantially constrained by the COVID-19 pandemic. Second, the Bayesian correlation analyses quantify the evidence and uncertainty associated with the pairwise within-participant relationships, while the bootstrap estimation and leave-one-out sensitivity analyses assess the robustness of the multivariable regression findings. However, these analyses do not compensate for the absence of independent participants or establish clinical generalizability. Third, one recording session contained high-frequency artifacts likely related to tactile stimulation; however, the principal findings were restricted to lower-frequency bands and therefore were unlikely to be substantially affected.

### 4.2. Methodological Limitations

First, although the 17 longitudinal sessions enabled the exploration of within-participant associations, they do not constitute independent participant observations and therefore provide no inter-individual statistical power. Accordingly, the findings are participant-specific and require replication in larger, clinically heterogeneous cohorts.

Second, embodiment and simulator sickness were assessed only at the end of each session; EEG and BCI-performance measures were therefore summarized at the session level to align their temporal resolution with the subjective measures. This approach precluded characterization of within-session theta and beta dynamics and prevented direct investigation of trial-by-trial relationships between neural activity and subjective experience. Although trial-level EEG analysis could characterize neural fluctuations across motor-imagery trials, these changes could not be directly related to subjective experience in the absence of corresponding trial- or block-level ratings.

Third, potentially relevant contextual factors, including medication use, sleep quality, fatigue, attention, emotional state, motivation, and general clinical status, were monitored qualitatively at the beginning and end of each session but were not quantified using standardized instruments. Although no persistent changes were identified, subtle fluctuations and their potential influence on neural activity and subjective experience cannot be excluded [12,13].

Fourth, because the dataset comprised repeated sessions from a single participant rather than independent observations, all statistical analyses should be interpreted as exploratory within-participant analyses. Temporal autocorrelation, gradual learning, adaptation, and fatigue may all contribute to within-subject dependency, meaning that standard regression assumptions (including independence of residuals) may not be fully satisfied. Although the principal regression findings remained robust following bootstrap estimation and leave-one-out sensitivity analyses, these procedures do not eliminate dependency among repeated measurements. Replication in larger longitudinal cohorts with multiple participants will be necessary to determine the generalizability of the observed associations. Accordingly, the present findings should be considered hypothesis-generating rather than confirmatory.

Lastly, the regression model included three predictors for 17 observations, which is statistically underpowered and may yield unstable coefficient estimates or overfitting. The model was selected based on theoretical relevance rather than for confirmatory inference, and the results should therefore be interpreted cautiously. Larger longitudinal samples will be required to determine whether the observed associations replicate across participants.

### 4.3. Future Directions

Future studies should seek to replicate these findings in larger longitudinal cohorts across multiple centres and different XR–BCI configurations while incorporating time-resolved EEG analyses and trial-level measures of embodiment. Experimental manipulation of multisensory conflict would further enable direct testing of the mechanistic hypotheses raised by the present findings. Future studies should also employ hierarchical or mixed-effects modelling to appropriately account for within-participant and between-participant variability in repeated-session datasets. Finally, the development of adaptive XR–BCI systems capable of responding to fluctuations in neural activity or user experience may provide new opportunities to optimise embodiment, minimise cybersickness, and improve BCI performance. Collectively, these approaches may clarify how neural state, sensory uncertainty, and motor imagery jointly shape embodiment and neurophysiological adaptation during XR–BCI use.

Our previous work demonstrated that cybersickness was the strongest factor associated with embodiment in healthy participants. The present findings extend this line of research by suggesting that simulator sickness may also be associated with sensorimotor cortical dynamics during XR–BCI use in a participant with chronic spinal cord injury. Although these studies addressed different populations and research questions, together they are consistent with the hypothesis that simulator sickness is associated with both the subjective and neurophysiological dimensions of XR–BCI interaction. Confirmation of this hypothesis will require larger prospective studies combining behavioural, neurophysiological, and computational approaches.

## 5. Conclusions

The present findings suggest that embodiment during XR–BCI use is characterised by a dual structure, comprising behavioural stability alongside structured neural and experiential fluctuations across repeated sessions. Sensorimotor beta activity was consistently associated with simulator sickness across Bayesian correlation, multivariable regression, bootstrap, and leave-one-out sensitivity analyses, whereas frontal theta activity was associated with the sense of embodiment. Together, these findings are consistent with partially distinct neurophysiological correlates of embodiment and simulator sickness during XR–BCI use.

## Figures and Tables

**Table 1:** Bayesian correlation analyses between the principal behavioural and neurophysiological variables.

Variables	Pearson r	BF_10_	95% CI	Kendall τ	BF_10_	95% CI
Sense of Embodiment—F3 Theta Activity	0.531	3.16	[0.036, 0.738]	0.369	2.55	[0.017, 0.606]
Simulator Sickness—C3–C4 Beta Activity	−0.522	2.88	[−0.768, −0.054]	−0.261	0.88	[−0.519, 0.075]
Acquisition Accuracy—Online BCI Performance	0.661	10.97	[0.201, 0.851]	0.521	12.28	[0.119, 0.730]
Sense of Embodiment—Simulator Sickness	0.298	0.57	[−0.184, 0.636]	0.148	0.42	[−0.173, 0.423]
Sense of Embodiment—C3–C4 Beta Activity	0.057	0.30	[−0.389, 0.475]	0.044	0.31	[−0.261, 0.336]
Online BCI Performance—C3–C4 Beta Activity	0.268	0.49	[−0.227, 0.624]	0.248	0.76	[−0.097, 0.514]

Note: Targeted Bayesian correlation analyses between the principal behavioural and neurophysiological variables. Pearson correlation coefficients (*r*) and Kendall rank correlation coefficients (τ) are presented together with their corresponding Bayes factors (BF_10_) and 95% credible intervals. BF_10_ values quantify the relative evidence for the alternative hypothesis over the null hypothesis.

**Table 2:** Multiple linear regression predicting C3–C4 beta activity.

Predictor	Standardized β	*t*	*p*	Bootstrap *p* (BCa)	VIF
Sense of Embodiment (SoE)	0.374	1.781	0.098	0.079	1.165
Simulator Sickness (SSQ)	−0.678	−3.303	0.006	0.007	1.116
BCI Performance	0.276	1.377	0.192	0.114	1.061

Note. Multiple linear regression predicting C3–C4 beta activity. Overall model: *F*(3, 13) = 4.495, *p* = 0.023; R^2^ = 0.509; adjusted R^2^ = 0.396. Bootstrap significance values were obtained using bias-corrected and accelerated (BCa) bootstrap estimation based on 5000 resamples. No evidence of problematic multicollinearity was observed (all VIFs < 1.2). Model diagnostics: Durbin–Watson = 2.423 (*p* = 0.431); maximum Cook’s distance = 0.586.

## Data Availability

The data are not publicly available due to patient confidentiality, privacy, and ethical restrictions. A description of all analysis scripts (OpenViBE scenario files) is available in a public repository (https://osf.io/9hmxj/overview?view_only=c74da968c4b443fe88502dc5304d474e) (accessed on 9 August 2026). The repository includes representative data and a detailed description of the BrainVision Analyzer preprocessing workflow sufficient to reproduce the analysis pipeline.

## Supplementary Materials

The following supporting information can be downloaded at: https://www.mdpi.com/article/10.3390/life16081352/s1. Figure S1. Representative raw and preprocessed EEG signals and power spectral density; Figure S2. Representative EEG activity during a single trial; Figure S3. OpenViBE processing pipeline used in the study; Figure S4. Offline EEG-processing workflow applied in the study.
