# Supplementary material for: Linking Embodiment, Simulator Sickness, and EEG Activity During XR–BCI Use: A Single-Participant Case Study
Source: Life (Basel). 2026 Aug 17;16(8):1352. doi: 10.3390/life16081352 (PMC13514828; doi:10.3390/life16081352)
Supplement: Supplementary file 1 [file life-16-01352-s001.zip › life-4444246-supplementary.pdf]

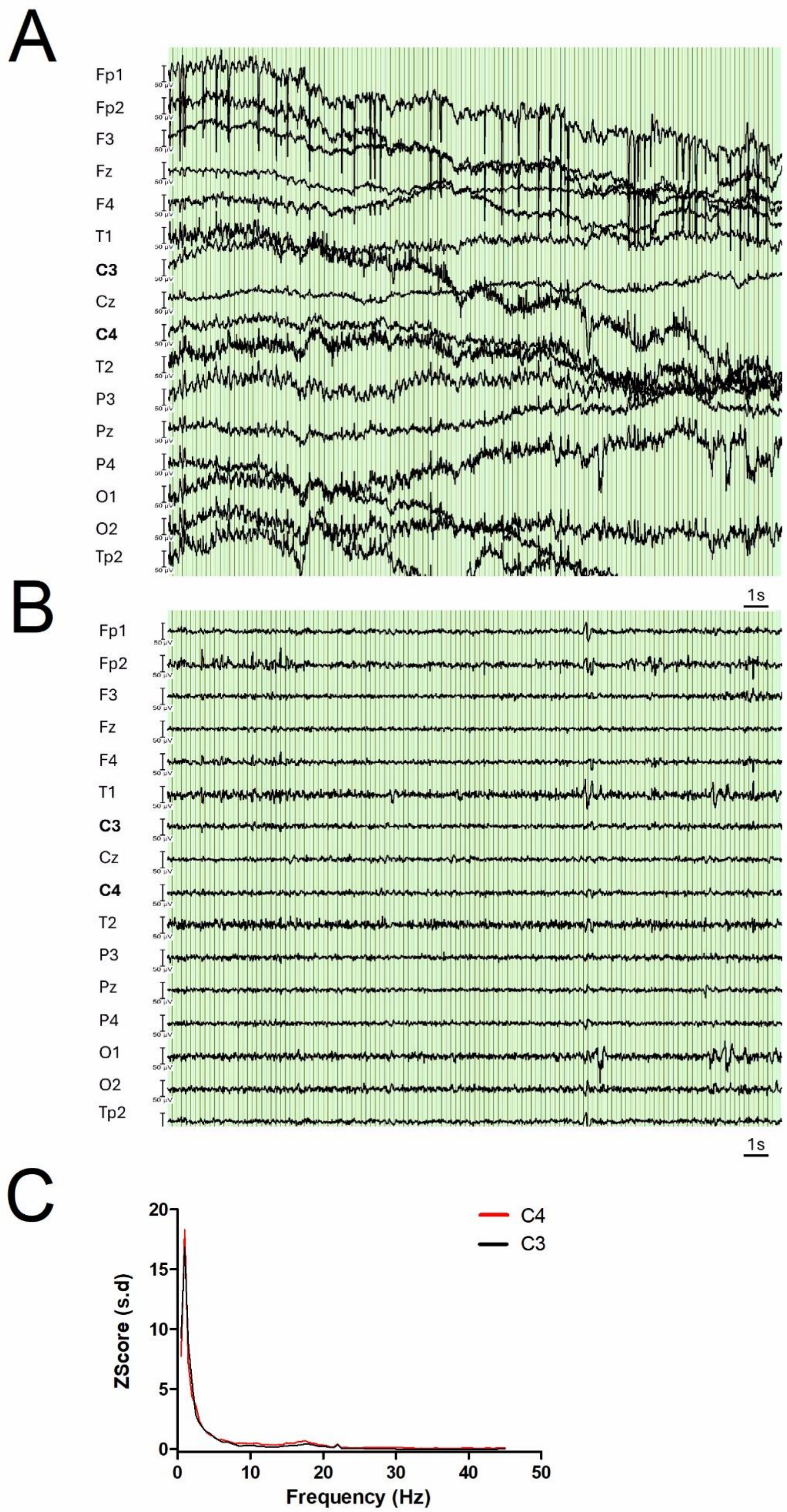

A
Fp1
Fp2
F3
Fz
F4
T1
C3
Cz
C4
T2
P3
Pz
P4
O1
O2
Tp2
1s
B
Fp1
Fp2
F3
Fz
F4
T1
C3
Cz
C4
T2
P3
Pz
P4
O1
O2
Tp2
1s
C
C4
C3
ZScore (s.d)
20
15
10
5
0
0
10
20
30
40
50
Frequency (Hz)

Supplementary Figure S1. Representative raw and preprocessed EEG signals and power spectral density. (A) Representative segment of raw EEG recorded from the 16 scalp electrodes. (B) The same EEG interval following preprocessing. The vertical calibration bars indicate 50 μV, and the horizontal bars indicate 1 s. (C) Power spectral density of the preprocessed C3 and C4 signals, calculated using the retained data from the complete recording session and expressed as z-score-normalized spectral power. Raw and preprocessed traces are shown for a representative interval to permit visual comparison, whereas the spectral estimates were obtained from the complete session to provide a more stable representation of the frequency content.

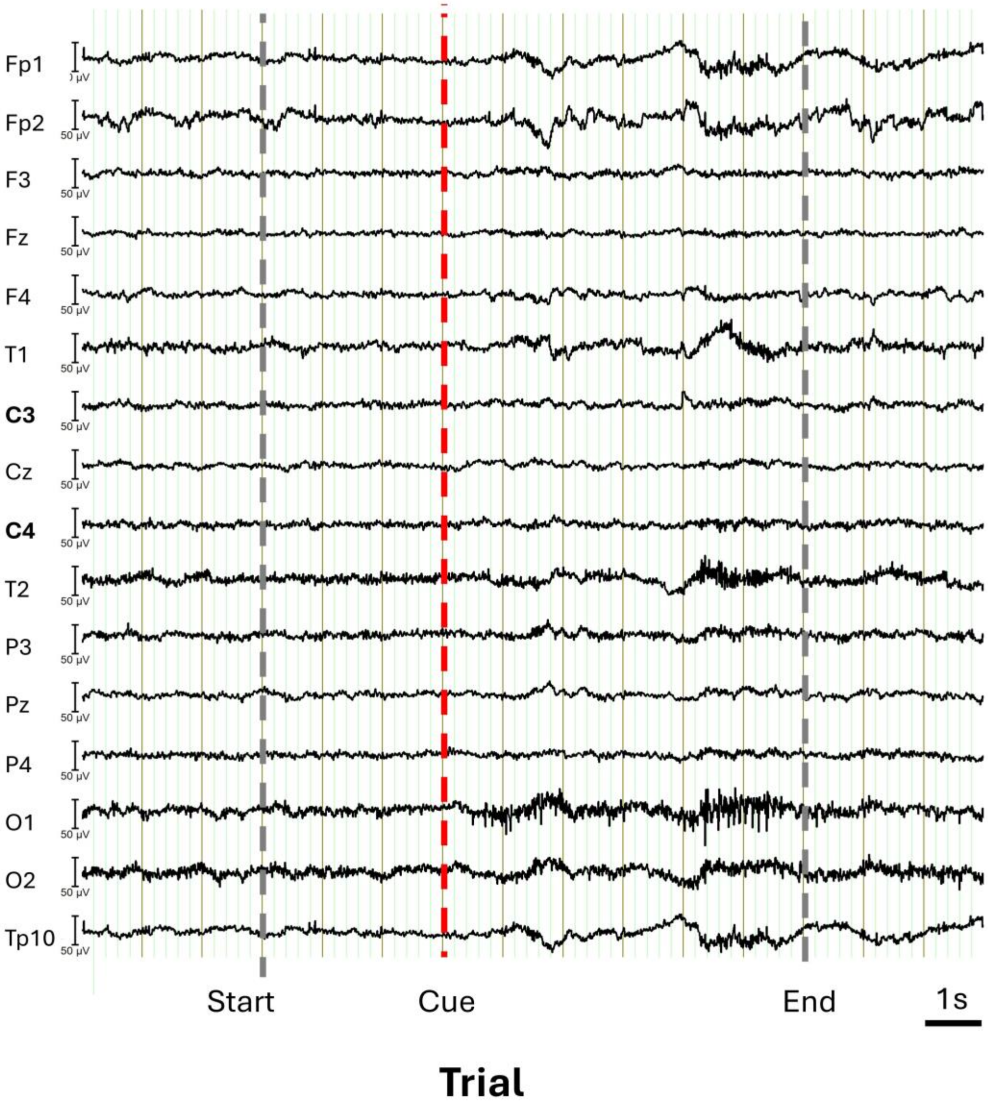

Supplementary Figure S2. Representative EEG activity during a single trial. Representative EEG traces recorded from the 16 scalp electrodes during one experimental trial. Vertical markers indicate the *Start*, *Cue*, and *End* segments of the trial, with the cue onset highlighted by a red dashed line. The vertical calibration bar corresponds to 50 µV, and the horizontal bar corresponds to 1 s. This example illustrates the temporal structure of the trial and the spatial distribution of EEG activity across the scalp during task execution.

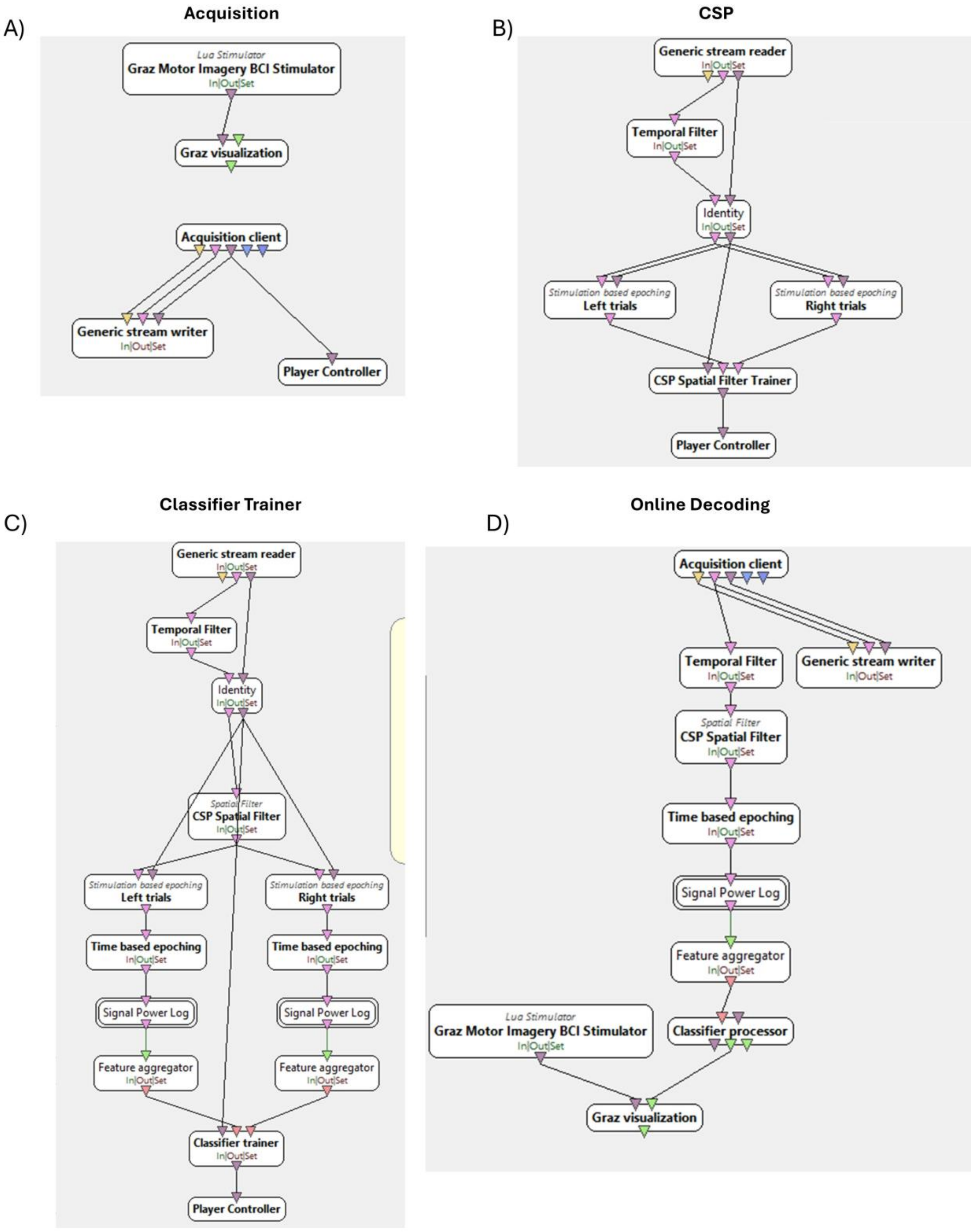

Supplementary Figure S3. OpenViBE processing pipeline used in the study. Panel A shows the acquisition setup; Panel B illustrates CSP spatial filter training; Panel C presents

the classifier-training workflow; Panel D depicts the online decoding chain integrating filtering, CSP, feature extraction, and real-time classification.

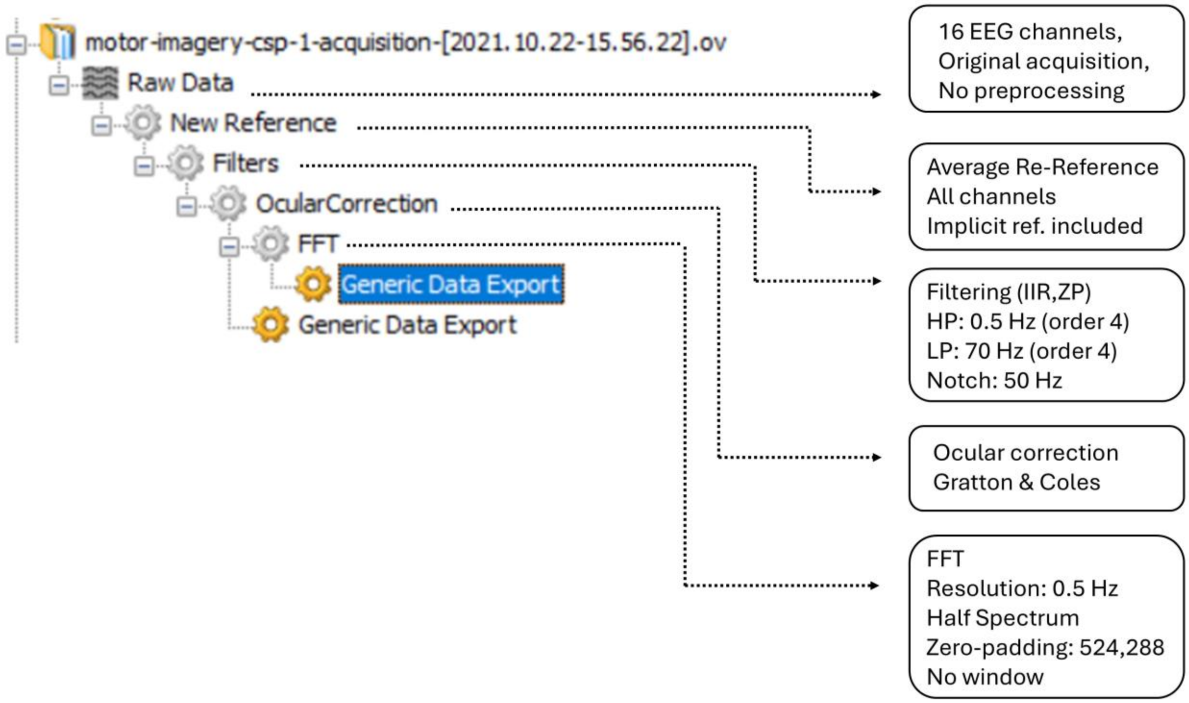

Supplementary Figure S4. Offline EEG-processing workflow applied in the study. The diagram summarizes the steps used in BrainVision Analyzer, including average re-referencing, IIR zero-phase filtering, ocular-artifact correction, and FFT-based spectral analysis prior to data export.
